# Quantification of Hydrogen Excess Adsorption in Silica Aerogels and Sandstone Using High-Pressure NMR T_2_ Relaxation

**DOI:** 10.1021/acsomega.6c06070

**Published:** 2026-07-09

**Authors:** Karol M. Dąbrowski

**Affiliations:** Faculty of Drilling, Oil and Gas, 49811 AGH University of Krakow, al. Mickiewicza 30, 30-059 Krakow, Poland

## Abstract

In this study, high-pressure NMR T_2_ relaxation is used to determine excess adsorption of hydrogen in two silica aerogel powders (hydrophilic and hydrophobic) and a Berea sandstone core over the range 1–350 bar. Hydrogen uptake is defined in terms of Gibbs excess adsorption, ensuring consistency with volumetric adsorption measurements at high pressure. The method incorporates calibration of NMR signal intensity using free-gas reference measurements and an independent calibration curve relating signal amplitude to gas volume. The hydrophilic aerogel exhibits the highest excess adsorption across the full pressure range, while the Berea shows intermediate behavior, exceeding the hydrophobic aerogel When normalized by mass, both aerogels demonstrate adsorption capacities orders of magnitude higher than the sandstone. These results confirm that T_2_-resolved NMR can provide a nondestructive method for quantifying excess hydrogen adsorption in porous media and highlight the influence of pore structure and surface chemistry on high-pressure hydrogen storage performance.

## Graphical abstract

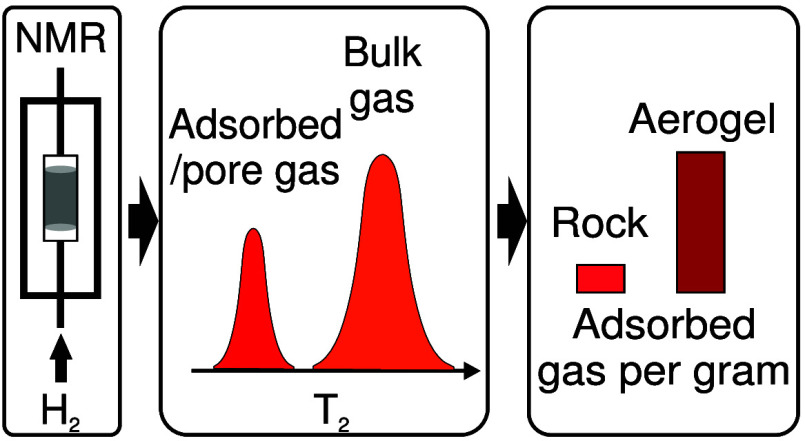

## Introduction

Hydrogen is a high–specific-energy, zero-carbon energy carrier with applications in mobility, power generation, chemical processing, and seasonal energy storage.^1,2^ Its large-scale deployment depends on storage systems that are safe, compact, reversible, and cost-effective. Conventional compressed-gas and cryogenic-liquid systems face inherent limits: compressed tanks offer only moderate volumetric efficiency, whereas cryogenic systems suffer from boil-off losses and high energy penalties.^3^ These constraints motivate the development of solid-state and subsurface storage strategies.^4,5^

Solid sorbents and geological formations offer promising pathways for hydrogen storage. Complex hydrides such as LiBH_4_ exhibit very high theoretical hydrogen densities, and additive modification (e.g., metal oxides or chlorides) can lower dehydrogenation temperatures and improve partial reversibility.^6^ Subsurface storage in depleted reservoirs, aquifers, salt caverns, and clay-rich seals^7^ relies on the partitioning of hydrogen between free gas and an adsorbed phase, which governs retention, leakage risk, injectivity, and working-gas fraction.

Adsorption increases hydrogen density locally at pore surfaces, enabling storage capacities higher than pure compressed gas. In porous carbons,^8^ clays,^9^ and shales,^10^ adsorbed hydrogen can reach densities several times greater than the bulk gas at identical pressure, substantially enhancing volumetric performance. Uptake scales with accessible surface area and microporosity; thus, controlling pore volume is essential. Engineered carbons, including carbon aerogels,^8,11,12^ can achieve 4–5 wt % at cryogenic temperatures through adsorption.^13^ Confinement in slit-shaped nanopores produces dense interfacial layers, an effect first illustrated by graphite nanofibers whose pore widths closely match the kinetic diameter of H_2_. Hydrogen adsorption also differs fundamentally from that of CH_4_ or CO_2_. Because H_2_ is weakly polarizable and possesses only a small quadrupole moment, its adsorption energy is significantly lower than that of larger, more polarizable gases. Consequently, the optimal pressure–temperature window for H_2_ adsorption shifts toward lower temperatures and higher pressures, and models developed for CO_2_/CH_4_ cannot be extrapolated reliably to hydrogen.^14^ Dedicated measurements and adsorption models are therefore required to describe its thermodynamics. Furthermore, hydrogen adsorption differs fundamentally from that of CO_2_ or CH_4_, not only in terms of interaction strength but also in the shape of excess adsorption isotherms at high pressure, where bulk-gas density effects become dominant.^15^

In real reservoirs, hydrogen rarely occurs alone. Co-present gases such as CO_2_ or CH_4_ and formation water/brine compete for adsorption sites. Water adsorbs strongly onto mineral surfaces, reduces available pore volume, and can suppress H_2_ uptake, while CH_4_/CO_2_ exhibit higher binding energies and preferentially occupy active sites. Understanding competitive and multicomponent adsorption is therefore essential for realistic assessment of subsurface storage performance and cushion-gas design. Silica-rich materials such as quartz and silica aerogels generally exhibit much lower hydrogen adsorption than carbons or clays due to their nonpolar surfaces,^16,17^ weaker van der Waals interactions, and limited polarizable sites.^18^ Studying silica systems, however, remains important because sandstone, silica aerogels, and siliceous shales constitute major fractions of reservoir rocks and engineered porous media.^19^ Their characteristically weak H_2_ affinity provides a baseline for comparison and helps identify when strong adsorption mechanisms (e.g., clay interlayers or carbon micropores) dominate hydrogen retention.^20^

Hydrogen adsorption can be quantified using several complementary methods. The volumetric Sieverts technique measures pressure changes in a calibrated manifold to obtain excess and absolute adsorption.^21^ Gravimetric sorption directly measures mass changes with a microbalance. Nitrogen adsorption at 77 K provides BET surface area and micropore volume, while DFT and Dubinin–Radushkevich analyses yield pore-size distributions.^22^ Low-field NMR distinguishes adsorbed and free hydrogen by relaxation times (T_1_, T_2_), allowing direct quantification of surface-bound hydrogen in geological materials.^23,24^ When combined with adsorption isotherms and molecular simulations (GCMC/MD), these methods link atomistic binding behavior to macroscopic storage performance.^20,25,26^

In this study, high-pressure NMR T_2_ relaxation is applied to quantify hydrogen adsorption in hydrophilic and hydrophobic silica aerogel powders and to compare them with Berea sandstone, enabling separation of adsorbed and free phases and revealing differences in pore structure and surface affinity.

At high pressure, adsorption measurements are commonly reported as Gibbs excess adsorption, which represents the amount of gas adsorbed in excess of the quantity that would occupy the same pore volume at bulk density. This distinction is particularly important for hydrogen, where the bulk-gas density increases rapidly with pressure, leading to a nonmonotonic behavior of excess adsorption isotherms. As a result, excess adsorption may decrease at high pressure even though the absolute amount of gas present at the surface continues to increase. Accurate interpretation of high-pressure adsorption data therefore requires explicit consideration of the excess adsorption framework.^15,27^

## Materials and Methods

### Samples

Three porous materials were investigated in this study: (i) a hydrophilic silica aerogel powder, (ii) a hydrophobic silica aerogel powder, and (iii) a Berea sandstone plug. A summary of their physical properties is provided in Table 1. The BET surface area and grain size values for the silica aerogels were obtained from manufacturer specifications, while the corresponding parameters for the Berea sandstone were taken from literature sources,^19^ as direct measurement under identical conditions was not feasible. The hydrophilic silica aerogel used in this work was the *Hydrophilic Silica Aerogel Powder from Ocellus*. This material possesses a naturally hydrophilic surface rich in silanol (−Si–OH) groups, resulting in a high affinity for polar fluids and a large specific surface area (see Table 1). Because the surface is unmodified, it retains its native water-wet character, which is relevant for the interpretation of gas–solid interactions in the NMR measurements. For comparison, a hydrophobic aerogel sample was included: *EnovaAerogel IC3105* from Cabot. The hydrophobic character of this aerogel arises from a TriMethylSilyl (TMS) surface treatment, where the original surface silanol (−Si–OH) groups are replaced by nonpolar methyl (−Si–CH_3_) functional groups. This substitution suppresses water adsorption, decreases moisture uptake, and enhances hydrophobic stability. Its relevant physical parameters are also listed in Table 1.

**Table 1:** Summary of Physical Properties of the Investigated Samples

sample	diameter (cm)	length (cm)	mass (g)	porosity (−)	BET (m^2^/g)	grain size (μm)	grain density (g/cm^3^)
hydrophobic aerogel	2.60	4.65	1.2	0.7057	600–800	100–500	0.13
hydrophilic aerogel	2.60	4.65	3.5	0.8502	600–800	150	0.2
Berea core (dry)	2.62	4.43	47.4	0.263	2.79	120–160	

A Berea sandstone 1” plug was included as a comparative natural porous medium and to validate the measurement workflow. In particular, the Berea sample was used to verify the experimental protocol, assess dead-volume effects, and provide a reference point against literature data for hydrogen behavior in low-surface-area geological materials. Its porosity was measured independently using a helium porosimeter, and its gas permeability was determined to be approximately 140 mD. The physical dimensions and grain characteristics are summarized in Table 1. For both aerogel powders, porosity values reported in Table 1 were computed from the measured sample volume, particle density, and total mass. Hydrogen gas (>99.95% purity) served as the working fluid for all experiments.

In addition to bulk properties, the investigated materials exhibit distinct pore morphology and surface characteristics. The silica aerogels are characterized by extremely high porosity and large specific surface area (600–800 m^2^/g), consisting predominantly of mesoporous networks with interconnected nanoscale pores. This structure provides a large number of surface sites accessible for gas adsorption. In contrast, the Berea sandstone is dominated by macropores and pore throats associated with intergranular void space, with relatively low specific surface area and limited microporosity. As a result, the available surface area for hydrogen adsorption is significantly lower than in aerogels, and gas storage is dominated by bulk pore filling rather than surface interactions.^19^

### NMR Instrumentation and Measurement Conditions

All NMR measurements were performed using a *GeoSpec 12/53 Rock Core NMR Analyzer* (Oxford Instruments), equipped with a 12 MHz permanent magnet. The spectrometer features a digital console with a programmable pulse sequencer, fast A/D acquisition, and a low-frequency optimized preamplifier capable of recording up to 128000 echoes. Radio-frequency transmission was provided by a Tomco amplifier with a peak power of 500 W. The system included a one-dimensional actively shielded pulsed-field gradient module (up to 20 G/cm), and the mains trigger allowed synchronization of gradient pulses with the AC line cycle. The magnet was fitted with an integrated temperature controller to maintain stable thermal conditions during the experiments. High-pressure experiments were conducted using an *Overburden P5* cell (Oxford Instruments) suitable for 1-in. samples and rated to 350 bar. Pressure control and modulation were achieved through a *Teledyne ISCO 100 DX* syringe pump. Echo trains were inverted to continuous T_2_ distributions using a multiexponential GIT SNR-based inversion algorithm over the T_2_ domain of 10^–2^–10^4^ ms, employing 121 logarithmically spaced values. A schematic of the complete gas-delivery and NMR measurement system, including gas regulation, high-pressure assembly, and sample handling components, is shown in Figure 1.

**Figure 1:**
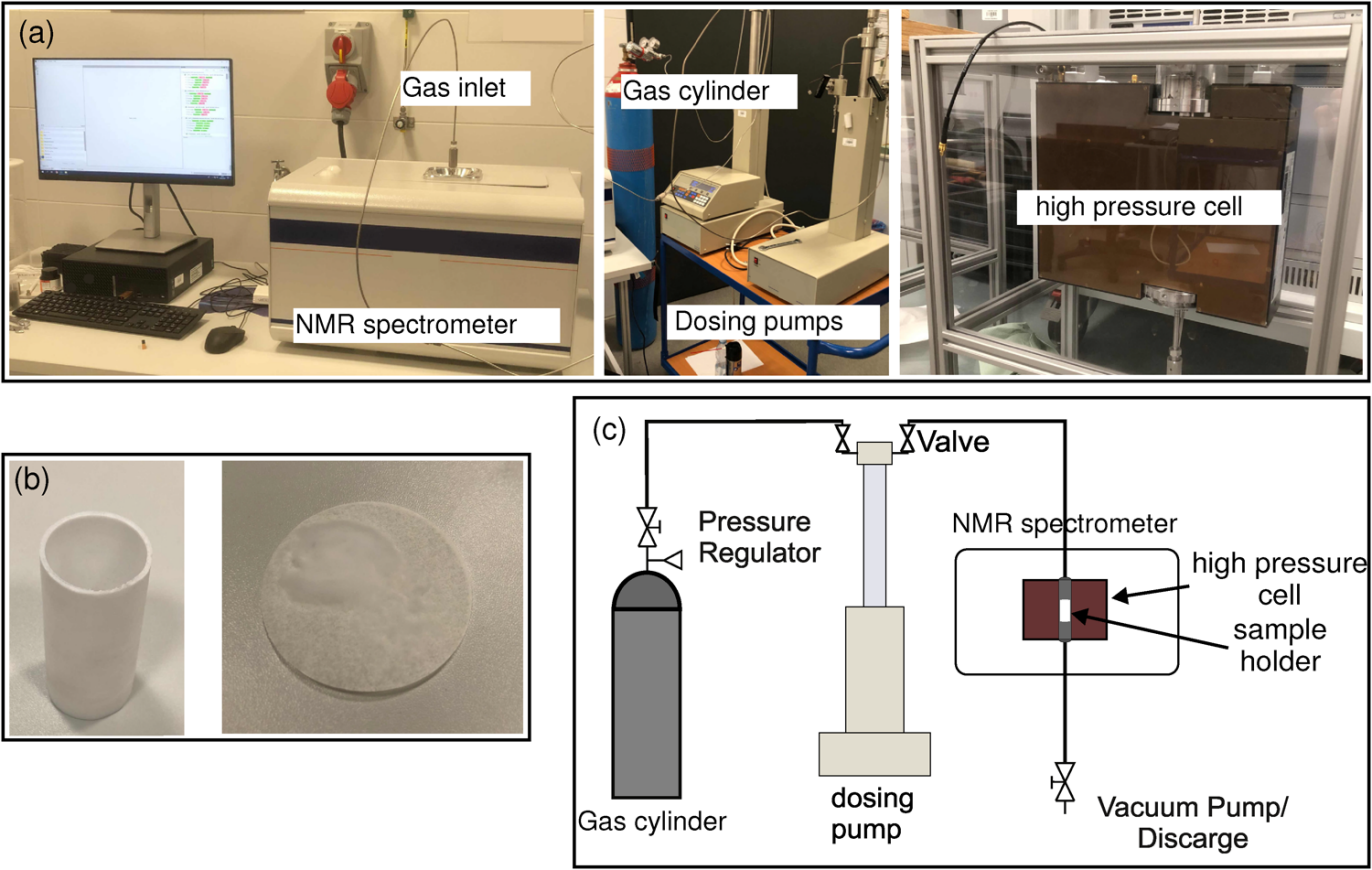
Schematic representation of the high-pressure gas-delivery and nuclear magnetic resonance (NMR) measurement system. (a) Integrated experimental layout comprising high-purity gas cylinders, dosing pumps, and the high-pressure cell interfaced with the *Oxford Instruments GeoSpec+* NMR spectrometer for in situ measurements under controlled thermodynamic conditions. Gas-supply and regulation module, including the primary gas cylinder, precision pressure regulator, and associated high-pressure lines enabling stable control of injection pressures during sample saturation. (b) Sample holder and aerogel powder sample (c) Detailed configuration of the high-pressure measurement assembly, containing the valve manifold, sample holder, high-pressure cell, dosing pump, and vacuum/discharge line used for controlled gas loading, evacuation, and pressure equilibration prior to NMR acquisition. All photographs were taken by the author.

### Extraction of Excess Adsorbed Hydrogen from T_2_ Distributions

Hydrogen in porous media exhibits distinct transverse relaxation behavior depending on its physical state. Gas occupying large pore spaces behaves as free or bulk gas and is characterized by long T_2_ relaxation times, whereas hydrogen experiencing strong surface interactions or geometric confinement exhibits shorter T_2_ relaxation.

In low-field NMR measurements, the short-T_2_ signal does not exclusively represent adsorbed hydrogen. Both surface-bound hydrogen and hydrogen confined within small pores exhibit enhanced relaxation and therefore overlap in the short-T_2_ region. As a result, the short-T_2_ signal reflects a combined contribution from adsorbed gas and gas in small pores. Due to the lack of chemical resolution, these contributions cannot be unambiguously separated and must be interpreted jointly.^28,29^

The T_2_ distribution is therefore partitioned into two regions using a cutoff value T_2,cut_, distinguishing hydrogen associated with confined and surface-dominated environments (short-T_2_) from free or bulk gas (long-T_2_). Rather than interpreting the T_2_ distribution as a direct volumetric function, the integrated NMR signal intensity in each region is treated as a proportional measure of hydrogen content.

The cutoff value T_2,cut_ used to separate short- and long-T_2_ components was set to approximately 1 ms. This value was determined from the observed separation between relaxation modes in the T_2_ distributions and supported by reference measurements, including empty-cell signals and samples with known pore characteristics. It should be noted that T_2,cut_ is not a universal constant, but depends on pore structure and fluid distribution, as discussed in previous studies.^30^ In low-field NMR, T_2_ is related to pore size through the surface-to-volume ratio (1/T_2_ = ρ_2_ *S*/*V*), and the cutoff is typically used as an empirical separation between surface-dominated and bulk-like pore environments. Repeated measurements confirmed that the position of the transition between short- and long-T_2_ components remains stable across the investigated pressure range, indicating that the chosen cutoff provides a consistent and reproducible partition of the NMR signal. Although the present study involves hydrogen gas rather than water-saturated systems, the same conceptual framework for interpreting T_2_ distributions applies.

The amount of free gas is calculated independently using the hydrogen density obtained from the Helmholtz equation of state

1 $$n_{\mathrm{free}}=\rho_{H_{2}}\left(p,T\right)\phi V_{\mathrm{bulk}}$$

Quantitative conversion of NMR signal amplitude to hydrogen content is achieved through calibration using known gas volumes (Figure 3(d)), establishing a linear relationship between signal intensity and hydrogen amount. This calibration accounts for differences in relaxation behavior between free and confined hydrogen and avoids direct interpretation of the T_2_ distribution as a physical volume function.

The hydrogen associated with the short-T_2_ region is then used as a measure of gas contained within surface-controlled environments, including both adsorbed gas and gas in small pores. Based on the calibrated signal, the corresponding gas amount is determined.

Finally, the Gibbs excess adsorption is calculated by subtracting the bulk-equivalent gas contribution occupying the same volume

2 $$n_{\mathrm{excess}}=n_{\mathrm{short}}-\rho_{H_{2}}\left(p,T\right)V_{\mathrm{short}}$$

This formulation follows the definition of excess adsorption, where the adsorbed amount excludes the gas that would occupy the same space at bulk density. At elevated pressures, this leads to the characteristic decrease in excess adsorption as bulk-gas density increases, even though the total amount of hydrogen within surface-controlled domains may continue to rise.

It should be emphasized that the short-T_2_ signal represents a combined effect of adsorption and pore-scale confinement, and therefore provides an effective measure of hydrogen associated with high surface-to-volume environments rather than a purely adsorbed phase. The calibration procedure and use of a reference sample (Berea sandstone) provide an internal validation of the methodology, compensating for the inability of low-field ^1^H NMR to directly measure nonproton-bearing gases such as CO_2_.

## Results and Discussion

Figure 2 shows the total amount of hydrogen occupying the pore volume of each sample, calculated directly from the Helmholtz equation of state and the independently determined pore volume. This quantity represents the free-gas content prior to any NMR-based phase separation and forms the basis for deriving the adsorbed fraction. When normalized by pore volume (Figure 2a), both aerogel powders store significantly more gas than the Berea sandstone due to their much larger porosities. The empty-cell signal provides a reference corresponding to the void volume of the NMR holder. When normalized by mass (Figure 2b), the contrast becomes even sharper: the aerogels show very high gas content per gram, reflecting their exceptionally low bulk densities, while the sandstone remains substantially lower across the entire pressure range. These total-gas trends illustrate how pore volume and bulk density govern the baseline amount of hydrogen available for subsequent partitioning into free and adsorbed phases.

**Figure 2:**
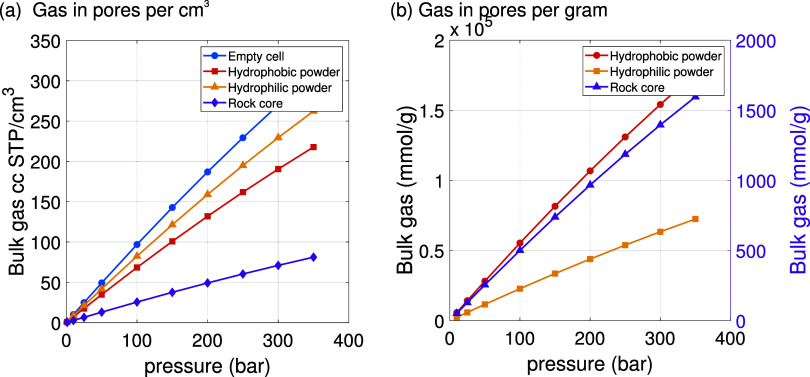
Hydrogen content in the pore space as a function of pressure, calculated from the Helmholtz equation of state using sample-specific pore volumes. (a) Total hydrogen normalized by pore volume (cc STP/cm^3^) for hydrophilic aerogel, hydrophobic aerogel, rock core, and empty cell. (b) Total hydrogen normalized by sample mass (mmol/g). No empty-cell value appears in panel (b), as the empty cell has no definable sample mass. The aerogels exhibit much larger mass-normalized gas content due to their extremely low bulk densities, while the sandstone shows markedly lower values. These plots provide the base free-gas quantities from which the adsorbed fraction is subsequently derived using NMR T*_2_* partitioning.

### T_2_ Distributions at Low and High Pressure

Figure 3(a,b) compares T_2_ distributions at 25 and 200 bar pressure, respectively. Signal form adsorbed gas (low T_2_) and signal from free gas is marked with colors. Signal was measured for hydrophilic, hydrophobic aerogels, Berea rock core and empty probe.

**Figure 3:**
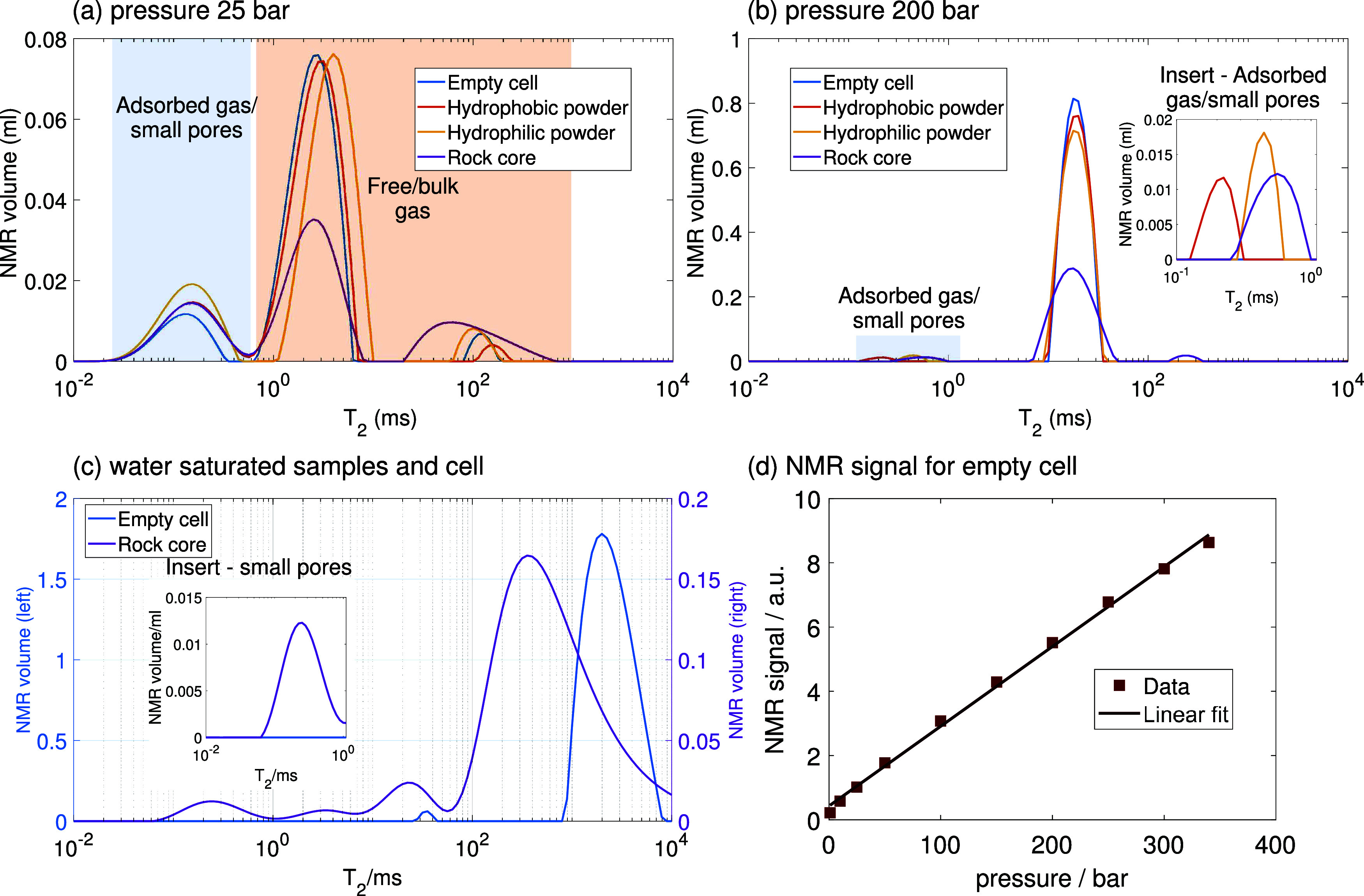
Transverse relaxation time (T_2_) distributions and calibration procedures for the investigated samples. Transverse relaxation time (T_2_) distributions for the investigated samples: (a) 25 bar and (b) 200 bar conditions, comparing empty cell, hydrophobic powder, hydrophilic powder, and rock core. The short-T_2_ region corresponds to hydrogen associated with surface and confined pore environments, while the long-T_2_ region represents free or bulk gas. (c) Water-saturated rock measurement and (d) NMR signal calibration used for quantitative analysis.

The figure presents T_2_ NMR relaxation time distributions for four sample types measured at two pressure conditions, 25 and 200 bar. The horizontal axis depicts the logarithm of the transverse relaxation time, spanning approximately from 10^–2^ to 10^4^ ms, while the vertical axis represents the corresponding NMR signal volume in milliliters. Each subplot includes measurements for the empty cell, a hydrophobic powder, a hydrophilic powder, and a low-porosity rock core. For both pressure conditions, the empty-cell measurements exhibit a pronounced long-T_2_ contribution associated with free gas occupying the void space of the measurement chamber. Notably, the amplitude of this long-T_2_ peak decreases at 200 bar relative to 25 bar. Since the shape and magnitude of the empty-cell distribution vary with pressure, a separate empty-cell measurement is required for each pressure. This ensures that the empty-cell contribution can be accurately subtracted from the total signal, thereby isolating the true gas response originating from the samples. Accurate subtraction of the empty-cell signal at each pressure is essential due to its pressure-dependent behavior. The short-T_2_ region corresponds to hydrogen associated with surface interactions and confined pore environments, including both adsorbed gas and gas in small pores, while the long-T_2_ region represents free or bulk gas. In this regime, both the hydrophobic and hydrophilic powders exhibit distinct peaks, indicating the presence of small pores/surface-bound gas. As discussed in the Materials and Methods section, these contributions cannot be separated explicitly using low-field NMR and are therefore interpreted jointly as hydrogen residing in surface-controlled environments.

The long-T_2_ regime, extending beyond approximately 10^2^ ms, is associated with free or bulk gas contained in pore spaces large enough to permit relatively unrestricted molecular motion. The hydrophilic and hydrophobic powder shows similar long-T_2_ signal to the empty cell, reflecting a high pore volume capable of storing significant quantities of free gas. The rock core, however, produces lower long-T_2_ response, in agreement with its characteristically smaller pore volume. An increase in pressure from 25 to 200 bar leads to a general rise in the total NMR signal for all samples, attributable to the higher gas density at elevated pressure. The short-T_2_ peaks for an empty cell is not present. For other samples a shift short-T_2_ peak can be seen. That shift results in interaction between hydrogen and material molecules. However, current NMR system is not capable to performed chemical resolved measurements therefore, that effects is solely qualitative. Type or energy of bounding cannot be determined. For the rock core, the overall signal remains lowest at both pressures, reaffirming its limited capacity for gas storage.

The water-saturated rock measurement (Figure 3(c)) shows that the water signal occupies the same short-T_2_ region as the confined hydrogen signal. The measurement was performed using a rock core saturated with water in an automated saturator (Vinci Technologies) and placed in an NMR cell completely filled with water. This reference configuration was additionally used to determine the dead volume of the measurement system. The overlap of water and hydrogen signals confirms that the short-T_2_ domain corresponds to hydrogen residing within the pore space, rather than representing a purely adsorbed phase. This demonstrates that both adsorption and geometric confinement contribute to the observed NMR response, highlighting that the short-T_2_ signal reflects hydrogen associated with high surface-to-volume environments.

The calibration curve (Figure 3(d)) shows a clear linear relationship between NMR signal amplitude and hydrogen content, confirming that signal intensity can be used for quantitative analysis. This calibration forms the basis for converting NMR signal contributions into gas amounts without relying on direct interpretation of the T_2_ distribution as a volumetric function.

### Kinetics at Fixed Pressure and Pressure-Dependent T_2_ Analysis

The NMR measurements shown in Figure 4(a,b) illustrate the time- and pressure-dependent behavior of hydrogen in a hydrophilic silica powder, expressed through transverse relaxation time (T_2_) distributions. Panel (a) presents the evolution of the T_2_ distribution at a fixed pressure of 200 bar over time (0, 39, and 89 min). An increase in the short-T_2_ signal is observed with time, accompanied by a concurrent increase in the long-T_2_ region. The growth of the short-T_2_ signal reflects an increase in hydrogen associated with surface and confined pore environments, including both adsorbed gas and gas in small pores. At the same time, the increase in the long-T_2_ signal indicates continued filling of larger pores and bulk gas regions. The total change in NMR signal over approximately 90 min is relatively small (around 5%), indicating rapid equilibration of hydrogen within the silica structure. This behavior is consistent with the high permeability and accessible pore network of silica-based materials, which allow fast redistribution of hydrogen between bulk and surface-controlled environments.

**Figure 4:**
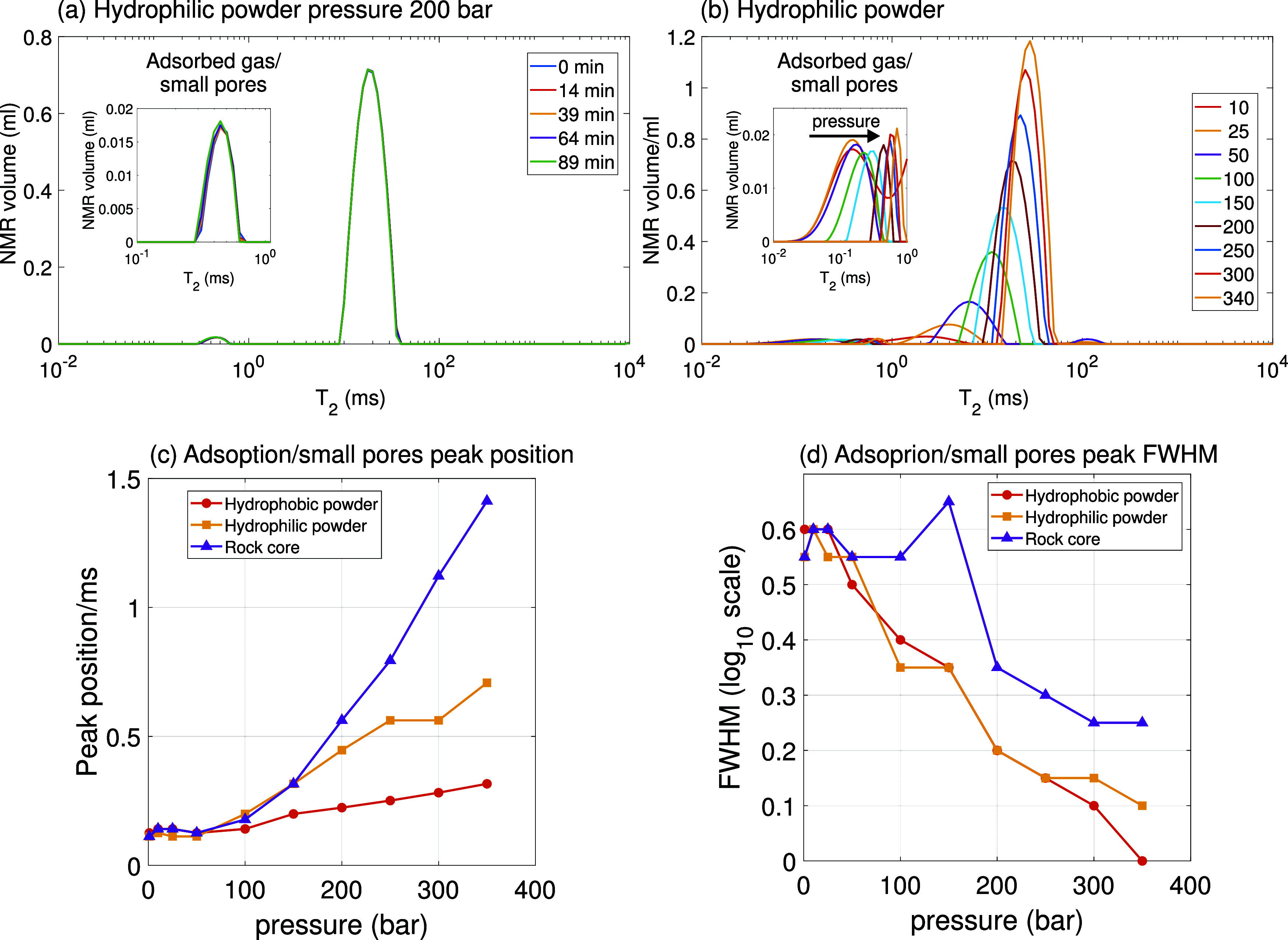
Time and pressure-resolved T_2_ relaxation distributions for a hydrophilic powder sample. (a) T_2_ distributions measured at a fixed pressure of 200 bar at different equilibration times (0, 39, 89 min), showing the evolution of adsorbed-gas and free-gas relaxation modes. (b) T_2_ distributions measured as a function of pressure (25–350 bar), illustrating how increasing pressure enhances the adsorbed-gas peak at short T_2_ and modifies the free-gas contributions. The NMR signal volume (mL) is plotted against log­(T_2_) for all experiments. (c) Pressure dependence of the adsorption-mode peak position. (d) Full width at half-maximum (FWHM) of the adsorption peak plotted in log_10_ space, highlighting broadening effects with increasing pressure.

Panel (b) shows the pressure-dependent evolution of T_2_ distributions over the range of 25 to 350 bar. With increasing pressure, both the short-T_2_ and long-T_2_ signals systematically increase, reflecting higher hydrogen density within the system. The increase in the short-T_2_ region indicates enhanced hydrogen accumulation in surface and confined pore environments, resulting from both adsorption processes and increased confinement effects at higher gas densities. In addition to amplitude changes, a shift of the short-T_2_ peak toward shorter relaxation times is observed with increasing pressure. This shift reflects increased interaction between hydrogen molecules and pore surfaces, as well as enhanced confinement effects due to higher occupancy of small pores. However, it should be noted that low-field NMR does not provide chemical specificity, and therefore the observed changes represent combined contributions from adsorption and geometric confinement rather than a uniquely defined adsorbed phase.

The long-T_2_ signal increases primarily due to the rise in bulk-gas density, which is governed by the thermodynamic behavior of hydrogen. Consistent with the calibration-based approach used in this study, the NMR signal amplitude reflects hydrogen content rather than pressure directly, and all quantitative interpretations are based on density-corrected values.

Overall, the evolution of the T_2_ distributions with time and pressure demonstrates the dynamic partitioning of hydrogen between bulk gas and surface-controlled environments, highlighting the combined influence of adsorption and pore-scale confinement effects on the NMR response.

Panels (c) and (d) quantify the pressure dependence of the short-T_2_ peak position and the corresponding full width at half-maximum (FWHM) on a log_10_ scale. The peak-position trends show that the rock core exhibits the largest shift of the short-T_2_ peak toward shorter relaxation times as pressure increases, followed by the hydrophobic powder, while the hydrophilic powder shows the smallest shift. This ordering reflects the differing strengths of surface interactions and pore-confinement effects across the three materials.

In contrast to the peak-position behavior, the FWHM in log-space decreases with increasing pressure for all samples. Both aerogel powders display similar narrowing trends and comparable peak widths, whereas the rock core consistently maintains a substantially broader peak, indicating a wider distribution of local environments and relaxation pathways within its more heterogeneous pore structure.

Although low-field NMR does not provide chemical specificity, the combined peak shifts and FWHM behavior indicate pressure-dependent changes in near-surface hydrogen density and pore-scale confinement. While the T_2_ distributions reliably separate adsorbed and free-gas populations, they report only relative phase proportions; absolute free-gas densities must be obtained through thermodynamic corrections rather than directly from NMR amplitudes.

### Pressure-Dependent Adsorption

The hydrogen content associated with surface-controlled environments was converted into excess adsorption as a function of pressure. In the present formulation, the NMR signal assigned to the short-T_2_ region represents hydrogen residing within high surface-to-volume environments, including both adsorbed gas and gas confined in small pores.

As shown in Figure 5, the excess adsorption increases with pressure in the low to intermediate pressure range for all investigated materials. This increase reflects the growing amount of hydrogen occupying surface-associated and confined pore regions as the bulk-gas density rises. At higher pressures, the excess adsorption exhibits a plateau or a decrease, particularly for the hydrophobic aerogel. This behavior can be further understood in the context of the Gibbs excess adsorption definition. The excess adsorption represents the difference between the amount of gas in the adsorbed phase and the amount that would occupy the same volume at bulk density. As pressure increases, the bulk gas density rises significantly, particularly under supercritical conditions. Consequently, the contribution of bulk-equivalent hydrogen within the adsorption space increases, leading to a reduction in the measured excess adsorption even though the absolute amount of hydrogen associated with the surface continues to increase.

**Figure 5:**
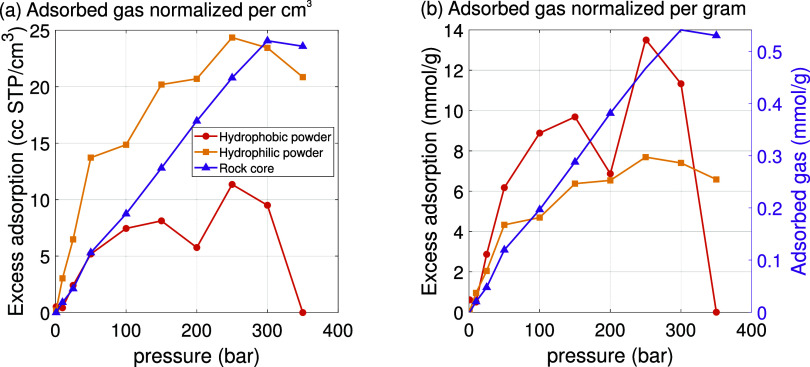
Pressure-dependent adsorbed gas content for hydrophobic powder, hydrophilic powder, and sandstone rock core over 10 bar 350 bar. (a) Adsorbed gas per unit pore volume (cc STP/cm^3^). (b) Adsorbed gas per unit mass (mmol/g). For panel (b), the left vertical axis corresponds to the powders and the right vertical axis to the rock core. The panels emphasize that powders exhibit higher adsorption per cm^3^ due to greater porosity, and much higher adsorption per g due to the combination of high porosity and very low bulk density; meanwhile, the hydrophobic powder can fall below Berea per cm^3^ at high pressure owing to supercritical Type III excess-adsorption behavior.

The more pronounced decrease observed for the hydrophobic aerogel reflects its weaker surface interactions with hydrogen. In this case, the density of hydrogen in surface-controlled environments approaches the bulk gas density more rapidly with increasing pressure, resulting in a stronger reduction in excess adsorption. In contrast, the hydrophilic aerogel, with a higher density of surface sites and stronger interactions, sustains a greater difference between surface-associated and bulk hydrogen densities, leading to a less pronounced decrease.

When normalized by pore volume (Figure 5a), the hydrophilic aerogel exhibits the highest adsorption across the entire pressure range, followed by the Berea sandstone, while the hydrophobic aerogel shows the lowest values. The hydrophilic sample displays a monotonic increase with pressure, followed by a gradual approach to saturation at higher pressures. In contrast, the hydrophobic aerogel shows a more pronounced plateau and a slight decrease at high pressure, consistent with excess adsorption behavior. The Berea sandstone exhibits intermediate values with a weaker pressure dependence, reflecting its lower surface area and adsorption capacity. When normalized by mass (Figure 5b), the trends differ significantly. Both aerogel powders exhibit substantially higher adsorption than the rock core, due to their low bulk density and high porosity. In this case, the hydrophobic aerogel shows higher adsorption than the hydrophilic aerogel across most of the pressure range. The hydrophobic sample again displays a more pronounced decrease at high pressure, while the hydrophilic aerogel shows a more gradual leveling-off behavior. The Berea sandstone remains significantly lower than both powders throughout the entire pressure range.

These contrasting trends highlight the strong influence of normalization choice (per pore volume versus per mass) as well as the combined effects of pore structure and surface chemistry on hydrogen adsorption behavior under high-pressure conditions.

The observed differences in hydrogen adsorption between aerogels and sandstone can be directly related to their pore structure and surface properties. The aerogels, with their high surface area and mesoporous structure, provide a large number of adsorption sites and enhanced surface interactions, leading to significantly higher adsorption capacity. In contrast, the Berea sandstone is characterized by larger pore sizes and lower surface area, resulting in weaker adsorption and a greater contribution from bulk gas. Consequently, hydrogen storage in sandstone is primarily governed by pore volume, while in aerogels it is strongly influenced by surface-controlled processes.

It should be emphasized that the NMR-derived signal used in this analysis represents hydrogen associated with surface interactions and pore confinement rather than a purely adsorbed phase. Therefore, the reported excess adsorption reflects hydrogen residing in surface-controlled environments, and its pressure dependence captures both adsorption and confinement effects within the porous structure.

It is important to note that the adsorption values obtained for the Berea sandstone are higher than those typically reported in the literature for similar materials. Recent studies have reported higher apparent adsorption values under conditions involving strong confinement or multicomponent systems. For example, Berea sandstone has been reported to exhibit excess adsorption on the order of 0.35 mmol g^–1^,^31^ while measurements of hydroxylated quartz as a sandstone model in binary gas mixtures of 0.9 mol hydrogen and 0.1 CO_2_ show mixture uptake approaching 0.5 mmol g^–1^ at elevated pressures.^32^

This discrepancy arises from the inherent limitations of the NMR-based methodology. In particular, the short-T_2_ signal used for quantification reflects not only truly adsorbed hydrogen, but also hydrogen confined within small pores and regions of restricted diffusion. As a result, the measured values represent hydrogen associated with surface-controlled environments rather than strictly defined adsorption. Additional contributions to the overestimation may arise from experimental factors such as uncertainty in dead-volume determination and the inability to use confining fluids in powder-based measurements. To ensure consistency across all samples, the same measurement approach was applied to both powders and the rock core, which may introduce a systematic bias. From this perspective, the reported adsorption values should be interpreted as an upper-bound estimate rather than a direct measure of thermodynamic adsorption. Importantly, although the absolute values are overestimated, the relative trends between materials remain robust and provide meaningful insight into the influence of pore structure and surface chemistry on hydrogen behavior. It should also be noted that several sources of uncertainty influence the quantitative extraction of adsorption values. The largest contributors include: (i) pressure-dependent variations in the empty-cell T_2_ distribution, necessitating independent empty references at each pressure; (ii) uncertainty in dead-volume determination within the high-pressure system; and (iii) contributions from hydrogen confined in small pores to the short-T_2_ signal. These factors collectively contribute to the systematic overestimation discussed above.

## Conclusions

Silica aerogels represent an effective model system for investigating hydrogen storage in porous media due to their extremely high porosity, tailored surface chemistry, and well-defined pore networks. The contrasting behavior of hydrophilic and hydrophobic aerogels enables systematic analysis of how surface affinity influences hydrogen uptake in surface-controlled environments under high-pressure conditions.

High-pressure T_2_ NMR provides a nondestructive framework for characterizing hydrogen distribution in porous materials. The reported values correspond to hydrogen residing in high surface-to-volume environments and are expressed in terms of Gibbs excess adsorption. The pressure dependence of hydrogen uptake follows the expected behavior of excess adsorption, with an initial increase at low and intermediate pressures followed by a decrease at high pressure due to the increasing bulk-gas density. This trend highlights the importance of distinguishing between excess and absolute adsorption when interpreting high-pressure data. It should be emphasized that the NMR-derived adsorption values represent hydrogen associated with surface-controlled environments and may overestimate the true thermodynamic adsorption capacity. Therefore, the results are most appropriately interpreted in a comparative sense rather than as absolute adsorption values.

The results confirm that sandstones, exhibit low hydrogen uptake, primarily due to their limited surface area and weaker surface interactions. When normalized by pore volume (cm^3^), the rock core exhibits adsorption values higher than the hydrophobic aerogel but lower than the hydrophilic aerogel, reflecting differences in pore structure and surface affinity. However, when normalized by mass (mmol g^–1^), both aerogels display significantly higher hydrogen uptake than the rock sample. In particular, the hydrophobic aerogel exhibits adsorption values up to approximately 25 times higher than the sandstone, while the hydrophilic aerogel reaches up to 15 times higher values. These pronounced differences arise from the extremely low bulk density and high accessible surface area of the aerogels. Silica aerogels therefore serve as useful benchmark systems for adsorption behavior in highly porous media. Looking forward, the combination of high-pressure NMR with controlled gas-exchange experiments offers a powerful approach for studying multicomponent systems relevant to underground hydrogen storage.

In particular, CO_2_ is a promising candidate for cushion-gas studies, as it produces no detectable signal in low-field NMR, enabling direct observation of hydrogen displacement, competitive adsorption, and hysteresis effects. Similar experiments with CH_4_ are also feasible, although interpretation requires additional corrections due to overlapping NMR signals. Overall, this study demonstrates that high-pressure T_2_ NMR provides a reliable platform for investigating hydrogen behavior in porous materials and for probing the combined effects of adsorption and pore-scale confinement. The approach establishes a framework for future studies of gas storage, transport, and displacement processes in both engineered materials and geological formations.

## Data Availability

All data and processing scripts will be made available upon request.
